# Symbiotic prokaryotic communities from different populations of the giant barrel sponge, *Xestospongia muta*

**DOI:** 10.1002/mbo3.135

**Published:** 2013-09-30

**Authors:** Cara L Fiore, Jessica K Jarett, Michael P Lesser

**Affiliations:** Department of Molecular Cellular and Biomedical Sciences, University of New HampshireDurham, New Hampshire, 03824

**Keywords:** Bacteria, sponge, symbiosis

## Abstract

The prokaryotic community composition of the ecologically dominant sponge, *Xestospongia muta*, and the variability of this community across in different populations of sponges from the Caribbean and Bahamas were quantified using 454 pyrosequencing of the 16S rRNA gene. The symbiotic prokaryotic communities of *X. muta* were significantly different than the surrounding bacterioplankton communities while an analysis of similarity (ANOSIM) of the sponge prokaryotic symbionts from three geographically distant sites showed that both symbiont and bacterioplankton populations were significantly different between locations. Comparisons of individual sponges based on the UniFrac *P*-test also revealed significant differences in community composition between individual sponges. The sponges harbored a variety of phylum level operational taxonomic units (OTUs) common to many sponges, including Cyanobacteria, *Poribacteria*, Acidobacteria, Chloroflexi, and Gemmatimonadetes, but four additional symbiotic phyla, previously not reported for this sponge, were observed. Additionally, a diverse archaeal community was also recovered from *X. muta* including sequences representing the phyla Euryarchaeota and Thaumarchaeota. These results have important ecological implications for the understanding of host–microbe associations, and provide a foundation for future studies addressing the functional roles these symbiotic prokaryotes have in the biology of the host sponge and the nutrient biogeochemistry of coral reefs.

## Introduction

Sponges comprise one of the most basal lineages of metazoans, dating back to *ca*. 600 million years ago (Bergquist 1978). Additionally, symbioses between prokaryotes and sponges are almost as old as the sponges themselves (Wilkinson 1984; Thiel et al. 1999). As a result the symbiotic prokaryotes of sponges have played a significant role in the evolutionary ecology and physiology of sponges (Thacker 2005; Fiore et al. 2010; Freeman and Thacker 2011). To better understand host–microbe evolution as well as the functional roles that sponges have in their ecosystems, additional taxonomic and functional characterizations of the sponge prokaryotic symbiotic community in a wide range of sponge species is needed.

It was first suggested by Hentschel et al. (2002) that there are widespread, sponge-specific microbial communities that are distinct from the surrounding environment. Furthermore, they hypothesized that there was a core community of prokaryotes common to all sponges, particularly high microbial abundance sponges (HMA, *sensu* Hentschel et al. 2006). Following this study, two subsequent analyses of publically available sequence databases confirmed the presence of many of the original sponge-specific clusters discovered (Taylor et al. 2007; Simister et al. 2012). The term “sponge-specific” was originally introduced to describe prokaryotic communities that have been repeatedly detected in sponges around the world but that differ from microbial communities present in the seawater (Hentschel et al. 2002; Taylor et al. 2007). In studies using the16S rRNA gene phylogenetic reconstruction is typically used to distinguish sponge-specific clades of prokaryotes within the complex sponge prokaryotic community (Hentschel et al. 2002; Taylor et al. 2007; Simister et al. 2012) while recent studies using high throughput sequencing have also utilized sequence similarity to determine whether or not sequences belong to a sponge-specific cluster of prokaryotic sequences (Taylor et al. 2013). The most recent assessment of sponge-specific sequence clusters has shown clusters within 14 bacterial phyla and within the Archaea as well as eukaryotic Fungi (Simister et al. 2012), however, many of the bacterial sponge-specific clusters described in the study by Simister et al. (2012) have recently been recovered outside of sponges (Taylor et al. 2013). Additional support for the occurrence of taxonomically similar prokaryotic communities from geographically distant sponges was recently reported in a metagenetic study using the 16S rRNA gene on 32 sponge species (Schmitt et al. 2011). Interestingly, this study also showed that there was evidence for a tropical clade of sponge microbes that are distinct from temperate and cold water sponge prokaryotic communities, potentially indicating the existence of subpopulations of sponge symbionts defined by environmental factors (Schmitt et al. 2011).

The most abundant groups present in sponges are of bacterial origin and include representatives from the phyla Actinobacteria, Chloroflexi, Proteobacteria, Cyanobacteria, Acidobacteria, and the candidate phylum *Poribacteria* (Taylor et al. 2007; Schmitt et al. 2011; Simister et al. 2012). Taxonomically, sponge prokaryotes of lower rank are also diverse, as the first pyrosequencing studies of sponge symbiont 16S rRNA genes revealed a higher number of operational taxonomic units (OTUs) than previously reported at the “species level” (Lee et al. 2010) and “genus level” (Webster et al. 2010). The use of high throughput sequencing methods to quantify symbiotic prokaryotic communities is increasing (Sogin et al. 2006; Uroz et al. 2010; Barott et al. 2012), largely due to the difficulty of culturing many prokaryotes and advances in sequencing technologies. The short reads (∼300 bp) generated by these next generation technologies have been shown to contain sufficient taxonomic information (Liu et al. 2007; Quince et al. 2009; Kunin et al. 2010) and can provide insight into rare members of sponge prokaryotic communities (Webster et al. 2010; Lee et al. 2010; Schmitt et al. 2011). However, full-length 16S rRNA gene sequences are still important for establishing phylogenetic “guide” trees (Ludwig et al. 1998; Taylor et al. 2007) and to populate the databases that unknown sequences can then be compared to.

The aim of this study was to understand and characterize the taxonomic variability of the prokaryotic community of the ecologically dominant giant barrel sponge, *Xestospongia muta*, (McMurray et al. 2008). *X. muta* is a prominent member on coral reefs throughout the Caribbean and Bahamas and when collected from the same depth and general conditions from multiple sites can be used to quantify and compare the taxonomic composition of the prokaryotic symbionts in different sponge populations as in a “natural experiment” (sensu Diamond 1986). Most of the previous work on *X. muta* has been conducted in one location (Florida Keys) and this study includes that site and from a comparative perspective expands our understanding of the prokaryotic communities associated with this important coral reef sponge. Furthermore, *X. muta* is known to harbor Archaea (Lopez-Legentil et al. 2010), a group increasingly recognized as having important roles in nutrient cycling (Hallam et al. 2006; Fiore et al. 2010; Hatzenpichler 2012), however, little is known about the taxonomic composition of Archaea in this sponge. We performed a metagenetic study that used pyrosequencing of 16S rRNA genes from *X. muta* samples collected from reefs near Key Largo, Florida (FL), Lee Stocking Island, Bahamas (LSI), and Little Cayman, Cayman Islands (LC). We hypothesized that not only will the sponge symbionts be significantly different from bacterioplankton populations in the overlying water column but there will also be location-specific differences in the prokaryotic community composition of *X. muta*.

## Experimental Procedures

### Sample collection

Replicate sponges (*n* = 3) were sampled at approximately 15 m from each of three locations: Rock Bottom Reef, Little Cayman, Cayman Islands (LC) (19°42′7.36″N, 80°3′24.94″W), North Perry Reef, Lee Stocking Island (LSI) (23°47′0.03″N, 76°6′5.14″W), Bahamas, and Conch Reef, Key Largo, FL (FL) (24°57′0.03″N, 80°27′11.16″W). All populations were sampled during the late spring and summer of 2011 where the maximum photosynthetically active radiation (PAR; 400–700 nm) irradiance at noon for these depths at all three locations is ∼500–600 μmol quanta m^−2^ sec^−1^ (M. P. Lesser, unpubl. data). Sponge pieces were cut from the top rim of the sponge (“pie slice” of pinacoderm and mesohyl) and placed in a plastic bag of seawater and placed on ice until reaching the laboratory. Each sponge sample was then placed in DNA buffer (20% dimethylsulfoxide, 0.25 mol/L ethylenediaminetetraacetic acid [EDTA], saturated NaCl, Seutin et al. 1991). All samples were kept frozen until reaching the University of New Hampshire where they were maintained at −70°C. Seawater samples (*n* = 3, 4 L each) were collected contemporaneously and filtered onto 0.22 μm filters (Whatman, Piscataway, NY) and frozen in DNA buffer.

### DNA extraction, polymerase chain reaction, and sequencing

Hexadecyltrimethylammonium bromide (CTAB) DNA extractions were performed on all sponge and seawater samples as follows. A section of each sponge sample that included both the outer pigmented layers and inner mesohyl of the sponge were cut into smaller pieces with a razor blade for processing. Filters were cut in half (half saved for later use) and also cut into smaller pieces for processing. Samples for both sponges and filters were placed in 600 μL of 2× CTAB mixture (Tris, pH 8.0 [0.0121 g/mL], NaCl [0.0818 g/mL], EDTA [0.00744 g/mL], CTAB [0.002 g/mL]) and homogenized with a pestle and with brief sonication. Proteinase k (5 μL of 20 mg/mL) was added and samples were incubated at 64°C for 3 h. Equal volume of chloroform was added to the samples followed by centrifugation at 12,000*g* for 10 min. The aqueous layer was transferred to a new tube and DNA was precipitated with equal volumes of 100% ethanol. The samples were spun again for 10 min followed by two washes with 70% ethanol, and then the pellet was allowed to dry before being resuspended in 30 μL of molecular grade water. Extractions were checked for quality and concentration using a NanoDrop spectrophotometer (2000c, Thermo Fisher, Waltham, MA). In some cases, a phenol:chloroform:isoamyl alcohol (25:24:1) extraction was performed to clean the samples. Briefly, the suspension was brought to 100 μL volume, and 1/10 volume of potassium acetate was added followed by equal volume of phenol:chloroform:isoamyl alcohol and vortexed for 5 sec. Samples were centrifuged for 2 min at maximum speed and the aqueous layer was transferred to a clean tube. Three volumes of 100% ethanol were added to each sample and vortexed for 5 sec, and then centrifuged for 10 min at maximum speed. The pellet was then washed twice with 500 μL of 70% ethanol (2 min of centrifuge in between), and then allowed to air dry.

The 16S rDNA of each sample was amplified and barcoded for multiplexed pyrosequencing using Titanium adapter sequences A (forward primer) and B (reverse primer), and a 10 bp barcode sequence added to the polymerase chain reaction (PCR) primers. Primers designed to amplify Bacteria and Archaea (hypervariable V6 region) were used, consisting of the forward primer U789F (5′-TAGATACCCSSGTAGTCC-3′) and the reverse primer U1068R (5′-CTGACGRCRGCCATGC-3′) (Baker et al. 2003, Lee et al. 2010). Three reactions of 25 μL were performed for each sample and pooled prior to electrophoresis. The PCR consisted of 0.25 μL of 50X Titanium Taq polymerase (Clontech, Mountain View, CA), 2.5 μL of 10X Titanium Taq buffer, 0.2 mmol/L dNTPs (Promega, Madison, WI), 0.4 μmol/L of each barcoded primer, and 25 ng of genomic DNA template. Reactions were performed with a Thermocycler (Eppendorf Mastercycler, Wesseling-Berzdorf, Germany) using the following protocol: initial denaturation for 5 min at 95°C, 26 cycles of 95°C for 30 sec, 53°C for 30 sec, and 72°C for 45 sec, followed by 6 min at 72°C. PCR products were then electrophoresed on a 1% agarose gel and purified with Qiaquick gel extraction kit (Qiagen, Valencia, CA). Samples were then purified with Agentcourt AMPure XP bead kit (Beckman Coulter, Danvers, MA) and quantified with a DyNA Quant 200 fluorometer (Hoefer, Holliston, MA) per manufacturer's protocol prior to combining all samples in equimolar concentration. Samples were pyrosequenced on the ROCHE/454 GS FLX+ platform (Roche, Branford, CT) at the University of Illinois W.M. Keck Center for Comparative and Functional Genomics (Urbana-Champaign, IL).

### Taxonomic assignment and diversity estimations of OTUs

The Quantitative Insights Into Microbial Ecology (QIIME) pipeline v 1.4 (Caporaso 2010) on the Amazon Elastic Compute Cloud (EC2) was used for all analyses except where noted. Sequences from sponge and seawater samples, as well as coral samples (Jarett 2012), were analyzed together up to the step of clustering into OTUs, following which, sponge and seawater samples were analyzed separately. Raw sequence reads were filtered for quality by discarding short reads (<200 bp), or reads with more than two mismatches with the primer sequence, or with ambiguous nucleotides, or with an average quality score less than 25. A custom Perl script based on the QIIME script “split_libraries.py,” was used to trim primers from the sequences, assign reads to their sample of origin (based on multiplex identifier tags), and reverse complement the reads originating from the B adapter (reverse reads) (changes from QIIME default: -l 300 –M 2 –b 10 -z). Trie clustering (QIIME team, unpubl. data, http://qiime.org) was used to collapse reads that are prefixes of each other into clusters and discard singleton reads. This technique has been used to rapidly and easily remove erroneous reads from pyrosequencing and provides an alternative “noise” removal method to denoising, which is computationally intensive (Behnke et al. 2010). The UCLUST algorithm (Edgar 2010) was used to cluster the remaining reads into OTUs at 97% similarity (settings: –max_accepts 20, –max_rejects 500, –stepwords20, and –word_length 12) and the most abundant sequences was selected as the representative sequence for each cluster. ARB (Ludwig 2004) was used to align representative sequences to the SILVA nonredundant reference database, release 108 (Pruesse et al. 2007) using the SINA plug-in. Aligned sequences were reformatted in QIIME and with custom Perl scripts for use in the QIIME pipeline. The alignment was filtered and poorly aligned sequences (at least 50 consecutive nucleotides without gaps), flagged as a potential chimeric sequence by ChimeraSlayer (Haas et al. 2011), or with significant BLAST (Altschul et al. 1990) matches (*E*-value <1 × 10^−10^ and at least 97% identity) to a custom database of likely contaminants (18S rDNA of demosponges, alveolates from SILVA reference database) were discarded.

Taxonomy was assigned to representative sequences using the Ribosomal Database Project (RDP) classifier and a minimum confidence cutoff of 0.8 (Wang et al. 2007) in QIIME. Assignments of “Root” or “Root:Bacteria” were rechecked by BLAST against the NCBI nr database, and was discarded if the top hit was not 16S rDNA. In QIIME, OTU tables were rarefied to equalize sampling depth across samples (step size = 100, total seqs = 7500, num-reps = 20), then the alpha diversity metrics of observed species, inverse Simpson diversity index, and Shannon diversity index were calculated and rarefaction curves were composed. The Chao1 metric was not used because it is based on the ratio of singleton to doubleton sequences and we removed singleton sequences. An approximately maximum likelihood phylogenetic tree was built using Fasttree 2 (Price et al. 2010) in QIIME and was used to calculate weighted and unweighted UniFrac distance values and perform the UniFrac Monte Carlo significance test (Hamady et al. 2009). To assess the similarity between microbial communities in the sponge or seawater samples from different locations, Bray–Curtis distance values were calculated based on rarefied OTU tables for nonphylogenetic diversity comparisons. Principle coordinates were then generated and used to create two dimensional plots, which were resampled by jackknifing using the lowest number of sequences per sample (*n* = 7648). Multidimensional scaling (MDS) and analysis of similarity (ANOSIM) were also performed on the OTU table (created in QIIME) in the program PRIMER v 6 (Clark 1993; Clarke and Gorley 2006). The OTU table was square root transformed prior to analysis and Bray–Curtis similarity metric was used. The *R-*values produced by pairwise comparisons in the ANOSIM is the best indicator of differences between groups and an *R* of 0.5 was used as a critical threshold with values equal to or higher than 0.5 indicating a difference between group means (Clarke and Gorley 2006).

Analyses of variance (ANOVA) and the G test of independence were each utilized to examine potentially significant OTUs between two sample types (e.g., seawater vs. sponge, LC sponges vs. LSI sponges) using the OTU category significance tool in QIIME. The use of ANOVA allows for the determination of whether OTU relative abundance is different between categories (i.e., sample type or location), while the G test determines whether the presence or absence of an OTU is associated with a category. Comparison of OTUs between locations and individual sponges for LC sponges was performed using manual curation of the OTU table from QIIME and the Venn diagram program Venny (Oliveros 2007). Only OTUs that were found in all three individuals at a given location were used to represent that location. OTUs that were further investigated following these comparisons were manually curated to make bubble plots.

### Phylogenetic analyses

Representative OTU sequences for specific taxonomic groups were selected for treeing. Sequences and their closest matches from GenBank using the blastn tool were aligned to the SILVA nonredundant SSU reference database (108) in ARB using the SINA plug-in and sequences were added to the tree using the parsimony quick add tool in ARB. The alignment was also used to build neighbor-joining and maximum parsimony trees in the program MEGA version 5 (Tamura et al. 2011) and the three treeing methods were compared based on tree topology. A neighbor-joining tree is presented here and nodes are marked that are consistent with the other treeing methods. Sequences resulting from pyrosequencing were submitted to the CAMERA (Cyberinfrastructure for Microbial Ecology Research and Analysis, http://camera.calit2.net/) website under project accession CAM_P_0000957.

Phylogenetic trees were also built using OTU sequences resulting from location comparisons and individual sponge comparisons using the OTU table from QIIME as described in the above section. Selected sequences were aligned using CLUSTAL W (Thompson et al. 1994) in MEGA v 5 (Tamura et al. 2011) and used to build neighbor-joining (not shown) and maximum likelihood trees in MEGA v 5 (Tamura et al. 2011).

## Results

### Phylogeography of *X. muta* prokaryotic communities using 454 pyrosequencing

Pyrosequencing of the 16S rRNA genes yielded 323 542 sequence reads (average read length 289 nucleotides). Following quality filter steps and removal of singleton reads there were 233 469 reads that were then clustered into OTUs at 97% similarity. A total of 1664 OTUs remained following removal of chimeric sequences and likely contaminants (i.e., 18S rRNA gene sequences, chloroplast sequences). A total of 407 OTUs were recovered from the sponge samples and included 17 phyla (Acidobacteria, Bacteroidetes, Chloroflexi, Cyanobacteria, Deferribacteres, Deinococcus-Thermus, Firmicutes, Gemmatimonadetes, Nitrospirae, Planctomycetes, Proteobacteria, Spirochetes, Verrucomicrobia, Crenarchaeota, candidate phyla *Poribacteria*, *TM7*, and *SBR1093*). Seawater samples yielded 1458 OTUs and included 27 phyla. Additionally, sponges contained 185 unique OTUs, while 222 OTUs were shared with seawater OTUs.

A rarefaction analysis revealed that seawater samples were approaching an asymptote at around 7000 sequences per sample but were not sufficiently sampled to capture the total diversity of the community (Fig. S1A). The sponge samples did reach an asymptote around 7000 sequences per sample based on rarefaction analysis, and had a range of 7648–14,247 sequences per sample (Table 1). Most of these sequences clustered into a few OTUs for both sponge and seawater samples (Fig. S1B). Up to 279 OTUs in one sponge sample were recovered, and up to 514 OTUs per water sample was recovered. The alpha diversity estimate of observed species is the number of unique OTUs and as expected, these were similar to the “species level” (97%) number of OTUs for each sample (Table 1) while the average number of observed species was estimated to be 201 for sponges and 342 for seawater samples. The Shannon and inverse Simpson diversity metrics showed the sponges to be more diverse than the water samples, and showed FL and LSI to be more diverse than LC for both water and sponge samples (Table 1).

**Table 1:** Sampling depth, number of OTUs, and the diversity estimate (based on a rarefied OTU table, *n* = 7500 sequences) for each sample.

Sample	Sampling depth	No. OTUs (97%)	Observed species	Shannon	Inverse Simpson
XmFL.1	11,595	244	231	6.4	0.98
XmFL.2	12,064	251	233	6.4	0.98
XmFL.3	12,818	256	238	6.4	0.98
XmLC.1	10,369	121	113	3.6	0.83
XmLC.2	8477	163	160	4.7	0.90
XmLC.3	7648	187	180	6.0	0.97
XmLSI.1	11,027	218	205	6.3	0.98
XmLSI.2	13,451	222	204	6.1	0.98
XmLSI.3	14,247	279	248	6.5	0.98
H_2_OFL.1	23,082	497	337	5.5	0.93
H_2_OFL.2	22,838	458	315	5.3	0.93
H_2_OFL.3	21,438	375	264	5.1	0.91
H_2_OLC.1	19,050	498	327	5.4	0.92
H_2_OLC.2	21,468	483	349	5.2	0.91
H_2_OLC.3	21,773	514	237	5.3	0.91
H_2_OLSI.1	26,961	576	357	5.6	0.94
H_2_OLSI.2	21,664	451	312	5.6	0.94
H_2_OLSI.3	19,920	437	314	5.6	0.94

Water samples are abbreviated H_2_O and sponge samples are abbreviated Xm. Samples are further labeled by location (FL, Florida Keys; LC, Little Cayman; LSI, Bahamas) and replicate number (1–3).

**Figure 1:**
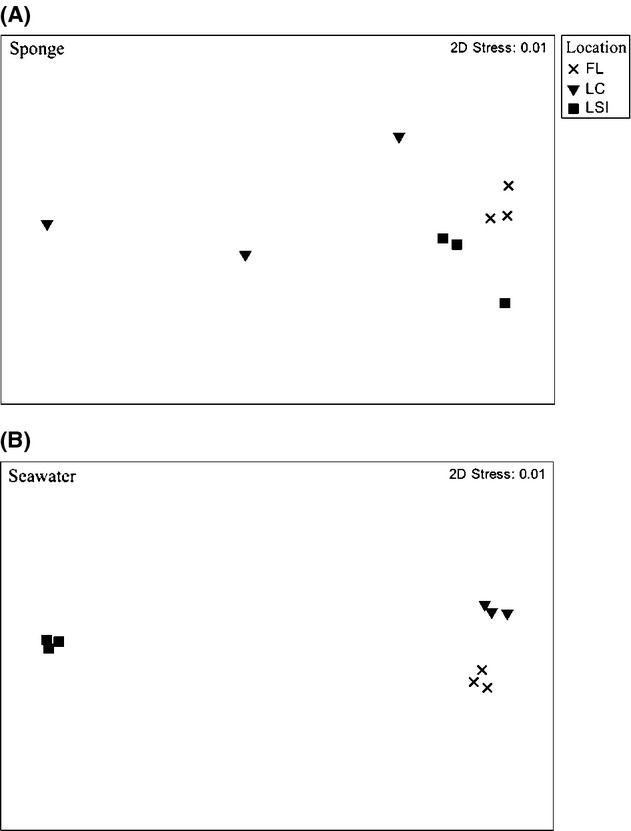
Multidimensional scaling analysis for sample location using Bray–Curtis similarity for the operational taxonomic unit (OTU) table (97% similarity) generated in Quantitative Insights Into Microbial Ecology (QIIME) (square root transformation) for sponge samples (A) and water samples (B). FL, Florida Keys; LC, Little Cayman; and LSI, Bahamas.

**Figure 2:**
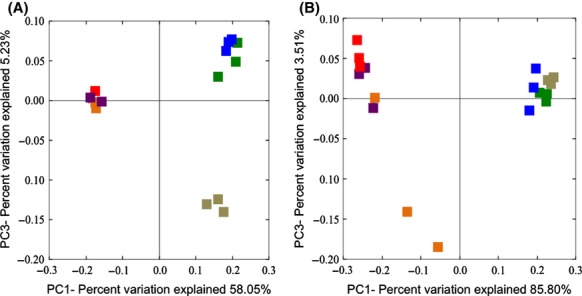
Principle-coordinate analysis plots based on weighted Unifrac distance comparing PC1 and PC2 (A) and unweighted Unifrac distance comparing PC1 and PC2 (B). Locations are color coded: red, XmFL; purple, XmLSI; orange, XmLC; blue, seawater FL; brown, seawater LSI; green, seawater LC.

The prokaryotic community composition of the seawater was significantly different from those of the sponges (Unifrac *P*-test, *P* < 0.05). MDS showed that the sponges grouped together by location and the same was observed for the water samples (Fig. 1). ANOSIM revealed a significant difference between locations for sponges and water, respectively (*R* = 0.5, *P* = 0.04 [sponges]; *R* = 1, *P* = 0.04 [water]), with significant differences resulting from pairwise comparisons between locations using the combined sponge or water samples from each location (*R* = 0.5–0.6, *P* = 0.1 [all sponges]; *R* = 1, *P* = 0.1 [all water]). Pairwise comparisons of individual sponges using the Unifrac p-test also yielded significant differences: XmLC1 is significantly different from XmFL1, XmLSI1, and XmLSI2 (Unifrac *P*-test, *P* < 0.05). Finally, XmLSI3 is significantly different from XmLC2 and XmLSI2 (Unifrac *P*-test, *P* < 0.05). Principle coordinates analysis (PCoA) showed clusters by location within sponge samples and within water samples to be more distinct with the weighted (by OTU abundance) than the unweighted Unifrac distance matrix (Fig. 2).

**Figure 3:**
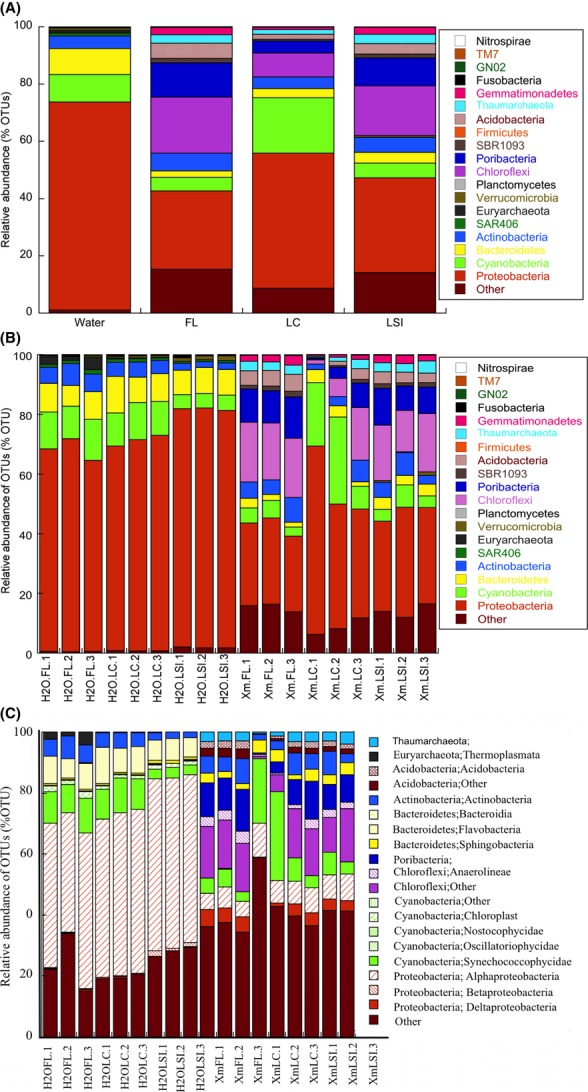
Average relative abundance of operational taxonomic units (OTUs) (97% similarity) at the phylum level for each sponge location and the water column (A), relative abundance of OTUs for individual samples (B), and relative abundance of OTUs (97% similarity) at the class level (as determined using RDP taxonomy) for each *Xestospongia muta* sample (1–3) at each location (FL, Florida Keys; LC, Little Cayman; LSI, Bahamas) and seawater samples at each location (C). The “Other” category in (B) contains unassigned Archaea, unassigned Bacteria, Chlamydia, PAUC34f, Spirochetes, ZB2 (seawater only), XB3 (seawater only), and Lentisphaerae (seawater only), which all had low relative abundance (<0.5%). The “Other” category in (C) contains unclassified Archaea, unclassified Bacteria, unclassified Bacteroidetes, Chlamydiae, two Firmicute classes, Fusobacteria, two GN02 classes, Gemmatimonadetes, Lentisphaerae, Nitrospira, PAUC34f, three Planctomycete classes, Epsilonproteobacteria, Gammaproteobacteria, one SAR406 class, two SBR1093 classes, two Spirochete classes, one TM6 class, two TM7 classes, one Tenericutes class, five Verrucomicrobia classes, ZB2, ZB3, and unclassified Proteobacteria.

Specific differences in relative abundance of OTUs between sponge and water samples were observed for several major groups including Proteobacteria, Choroflexi, Poribacteria, and Gemmatimonadetes (Fig. 3A). Within the individual sponge samples, the most obvious difference was that LC sponges generally harbored more cyanobacterial and proteobacterial OTUs and fewer OTUs within the Chloroflexi than sponge samples from other locations (Fig. 3B). Comparison of class-level taxonomy highlighted even more differences between sponge and water samples (Fig. 3C). For example, the OTUs classified as Proteobacteria in the water samples were almost all classified as Alphaproteobacteria, while in the sponge samples they were roughly split between Alpha- and Deltaproteobacteria. Also, many of the OTUs classified as Bacteroides from the water samples were identified as Flavobacteria while Bacteroides OTUs from sponges were dominated by Sphingobacteria. Lastly, differences in classification of archaeal OTUs at the phylum and class level were also observed between sponge and water samples (Fig. 3B, C).

The OTU category significance test in QIIME identified OTUs that were significantly more abundant in one group of samples than another group (Table S1). Of particular note, OTUs identified as more abundant in sponges relative to seawater and particularly so in FL and LSI sponges, included members of the *Poribacteria*, Acidobacterium, Syntrophobacteraceae, Entotheonellaceae, Gemmatimonadetes, Chromatiales, and Chloroflexi-4. In addition, OTUs identified as more abundant in sponges, but particularly in LC samples, included taxa of Synechococcaceae and Alphaproteobacteria. Further investigation into OTUs that are unique to each location revealed only one unique OTU for LC, 22 for LSI, and 46 for LSI. Comparison of the ten most abundant of these OTUs at each location revealed that OTUs with the highest abundance were from LSI sponges (Fig. S2). Additionally, unique OTUs from FL were largely comprised of Gammaproteobacterial OTUs, while other diverse groups were represented in the LSI OTUs, and one unidentified bacterial OTU was present in the LC sponges (Fig. S2).

**Figure 4:**
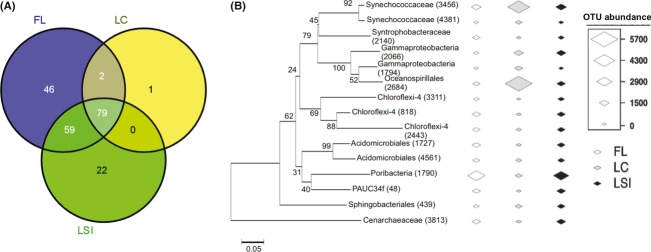
Comparison of OTUs between sponges at each location. Only OTUs that were found in all three individuals at a given location were used to represent that location. A Venn diagram showing overlapping operational taxonomic units (OTUs) between sponges at each location (A), and a maximum likelihood tree and corresponding bubble plot showing OTU abundance (B). Taxonomic names shown are the lowest taxonomic assignment by RDP. Bootstrap (*n* = 1000) values for the tree are shown and the scale bar represents 5% sequence divergence. “Bacteria” indicates that an unidentified bacteria was the closest match. Locations are represented by colors shown by the legend and a second legend provides a scale for OTU abundance. The OTU number (identity) is shown in parentheses for each OTU, and OTU abundance refers to the number of sequences within that OTU. FL, Florida Keys; LC, Little Cayman; LSI, Bahamas.

**Figure 5:**
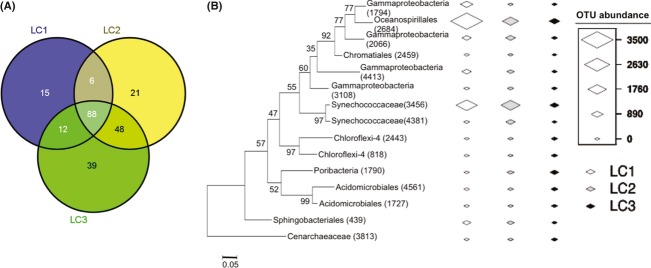
Comparison of operational taxonomic units (OTUs) between the LC sponges. A Venn diagram showing overlapping OTUs between individual sponges (A), and a maximum likelihood tree and corresponding bubble plot showing OTU abundance (B). Taxonomic names shown are the lowest taxonomic assignment by RDP. “Bacteria” indicates that an unidentified bacteria was the closest match. Bootstrap (*n* = 1000) values for the tree are shown and the scale bar represents 5% sequence divergence. Individual sponges are represented by colors shown by the legend and a second legend provides a scale for OTU abundance. The OTU number (identity) is shown in parentheses for each OTU, and OTU abundance refers to the number of sequences within that OTU. FL, Florida Keys; LC, Little Cayman; LSI, Bahamas.

The similarities and differences in the taxonomic composition of sponges between the three locations were visualized using a Venn diagram (Fig. 4) and showed that most OTUs are shared between all three locations. There are also many OTUs that are shared only between FL and LSI, but few or none that are shared between FL and LC and between LC and LSI (Fig. 4A). Comparison of the 15 most abundant OTUs that are shared between all three locations shows that FL and LSI have similar abundances of many of the shared OTUs (Fig. 4B). LC was investigated further because it was the most dissimilar of the three locations in terms of OTU abundance (Fig. 4). Most of the OTUs were shared between the three LC sponges, with individuals LC1 and LC2 sharing the fewest OTUs (Fig. 5A). The greatest differences in the abundance of shared OTUs are between sponges LC1 and LC3 (Fig. 5B). Investigation into unique OTUs within the LC sponges showed LC3 to have OTUs with higher abundance relative to LC1 and LC2 (Fig. S3). It is important to note that LC3 is the only sponge with unique poribacterial and archaeal OTUs, however, all three individuals have unique OTUs that are from diverse taxonomic groups (Fig. S3).

### Taxonomic composition of archaea in *X. muta*

**Figure 6:**
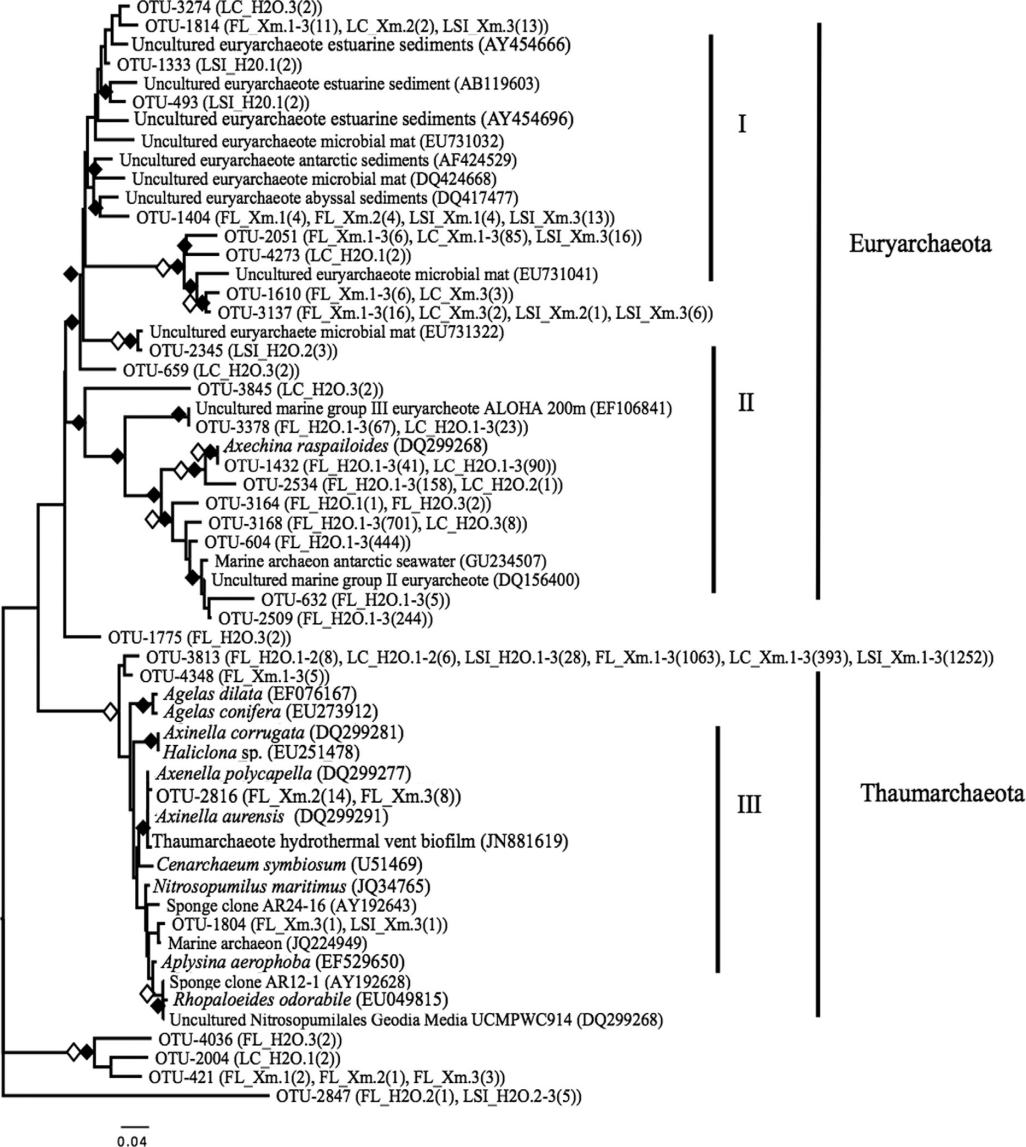
Neighbor-joining tree based on archaeal 16S rRNA genes of operational taxonomic unit (OTU) sequences from sponge and water samples. Filled in diamonds represent nodes also observed in the maximum parsimony tree and open diamonds represent nodes also observed in the ARB tree following addition of OTU sequences to the SILVA nr SSU dataset. The scale bar represents 4% sequence divergence.

Representative sequences from OTUs classified as Archaea were recovered from sponge and seawater samples and fell into three distinct clades (Fig. 6). Clades I and II were comprised of sequences representing the phylum Euryarchaeota. Clade I contained OTU sequences from both water samples and *X. muta* samples and were related to euryarchaeotes recovered from sediments and microbial mats. Clade II contained only OTU sequences from water samples and was most closely related to marine group II and III euryarchaeotes, and included one sequence from the sponge *Axechina raspailoides* (Fig. 6). Clade III contained only OTU sequences from *X. muta* samples, and these were related to the previously described thaumarchaeotes *Nitrosopumilus maritimus* and *Candidatus Cenarchaeum symbiosum* of the group I.1a Archaea. Some nonsponge-derived sequences from other datasets were also included in clade III, preventing it from being classified as a sponge-specific cluster. Other OTU sequences from sponge and water samples in the current study fell into the clade of Thaumarchaeota but were distinct from the clade III group of mostly sponge-derived sequences (Fig. 6).

## Discussion

The results of this study show that *Xestospongia muta* harbors a prokaryotic community that is distinct from the surrounding seawater, and that there is a significant effect of location for both the symbiotic prokaryotic community of the sponges and the bacterioplankton community. While the community composition overall was similar to that documented by Montalvo and Hill (2011), four additional phyla (Thaumarchaeota, Euryarchaeota, Spirochetes, and the candidate phylum *Poribacteria*) were recovered from *X. muta* in the current study, and one phylum (candidate phylum *TM6*) was only recovered from seawater in the current study. This supports previous findings that the 300–400 bp products generated using 454 pyrosequencing have sufficient taxonomic information for diversity studies because of the coverage across hypervariable regions and the development of appropriate bioinformatic approaches (Liu et al. 2007; Quince et al. 2009; Kunin et al. 2010).

Diversity estimates for *X. muta* in this study were similar to the diversity estimates reported previously (Montalvo and Hill 2011). The Shannon and inverse Simpson diversity metrics showed that sponge samples were more diverse than seawater samples. Diversity indices take into account both the number of unique OTUs and the number of sequences in each OTU. Therefore, the lower overall diversity of seawater samples, despite the greater number of OTUs, is a consequence of fewer sequences in those OTUs when compared to sponges who have fewer OTUs that are populated with significantly greater numbers of sequences, as further demonstrated by the steeper rank abundance curve (Fig S1B). The dominant phyla recovered from *X. muta,* based on relative abundance of OTUs, are similar to those recovered from studies on sponges in diverse locations and habitats (Taylor et al. 2007; Simister et al. 2012), including representatives from the Proteobacteria, Chloroflexi, Cyanobacteria, and *Poribacteria*. Additionally, OTUs classified as Actinobacteria, Bacteroidetes, Acidobacteria, Gemmatimonadetes, and Thaumarchaeota also contributed to the diversity of the symbiotic prokaryotic community composition in *X. muta*.

All of the major groups of prokaryotes associated with sponges, including Archaea, have been shown to contain monophyletic sponge-specific clusters (Simister et al. 2012). However, a recent study that examined sequences from environmental samples for the presence of sponge-specific clusters of bacteria found almost half of the previously described sponge-specific sequences (Simister et al. 2012) could be recovered from the environment (Taylor et al. 2013). The abundance of these sponge-specific sequences was very low, but as the authors suggest, the presence of putative sponge-specific clusters of bacteria outside of sponges indicate the existence of other potential reservoirs of sponge-associated bacteria (Taylor et al. 2013). Over half, however, of the original sponge-specific clusters are still considered sponge-specific (Taylor et al. 2013). For the sponge-specific clusters detected outside of sponges we do not know if they are metabolically active or if they are transient in the environment. In the present study, we use the term “sponge-specific” to include only those groups generally considered to be sponge-specific following the analysis of Taylor et al. (2013). This includes clusters within the Acidobacteria, Actinobacteria, Chloroflexi, Cyanobacteria, Gemmatimonadetes, Alphaproteobacteria, and Gammaproteobacteria. Archaeal sequences were not included in the study by Taylor et al. (2013), and we refer to previous phylogenetic studies when considering sponge-specific clusters within this group (i.e., Simister et al. 2012).

In a study comparing the prokaryotic community of the congenerics *X. muta* from the Florida Keys and *X. testudinaria* from the Pacific, some species-specific clusters within the Chloroflexi, Acidobacteria, and Cyanobacteria were observed based on clone libraries of the 16S rRNA gene (Montalvo and Hill 2011). This suggests some specificity of the symbiotic community at the genus level for the host. *Poribacteria* and Archaea, however, were not recovered from *X. muta* or *X. testudinaria* (Montalvo and Hill 2011), potentially due to bias issues (Suzuki and Giovannoni 1996) in the eubacterial primers (27F/1492R) used by Montalvo and Hill (2011). *Poribacteria* was considered a sponge-specific group, although sequences representing this group have also been documented in low abundance from other environmental samples (Webster et al. 2010; Taylor et al. 2013). Recently, the *Poribacteria* genome was characterized using single-cell genomics, providing insight into the functional role of this ubiquitous group of bacteria (Siegl et al. 2010). Genome characterization revealed a mixotrophic lifestyle for the *Poribacteria*, with the potential for carrying out denitrification, and the authors suggest that these are commensal bacteria within the sponge (Siegl et al. 2010). The *Poribacteria* are also one of the most diverse groups of sponge symbionts (Schmitt et al. 2011) and was a relatively diverse group (11 OTUs, 9377 sequences) in the current study.

Comparison of the bacterial symbiotic community composition in several sponges, including *X. muta*, over a depth gradient (9–90 m) showed that while there was some variability in the community over depth, each sponge maintained a “core” group of bacteria over the depth range (Olson and Gao 2013). Based on the differences in the symbiotic community of *X. muta* observed in this study, it would be informative to see if a “core” community is maintained over the shallow to mesophotic depth range at different locations and if the differences observed in the current study also occur over this depth range at these locations. With the growing realization of the importance of mesophotic reefs as a potential refuge and population source for their shallow water counterparts (Lesser et al. 2009), a better understanding of the symbiotic communities of ecologically important taxa, such as sponges, at mesophotic depths is needed.

Archaeal sequences recovered from *X. muta* were classified as Euryarchaeota or Thaumarchaeota, groups now commonly represented in sponge prokaryotic 16S rRNA gene libraries (Lee et al. 2010; Simister et al. 2012; Fan et al. 2012). Sponge-specific clusters have been documented for both phyla, although the Thaumarchaeota has received more attention as it contains the prevalent sponge symbiont *C. symbiosum* (Schleper et al. 1997; Hallam et al. 2006). The Thaumarchaeota also contain ammonia-oxidizing archaea (AOA), the discovery of which has had a significant impact of our understanding of marine nitrogen cycling (Herndl et al. 2005; Fiore et al. 2010; Hallam et al. 2006). Interestingly, the physiology of AOA (such as *Nitrosopumilus maritimus*) and what are now considered *amo*A-encoding archaea (AEA), such as *C. symbiosum* (Hatzenpichler 2012), is not well understood and more genetic and physiological studies are necessary to elucidate the mechanisms and regulation of ammonia oxidation in these organisms (Hatzenpichler 2012). A recent study specifically examining the archaeal community in sponges near Brazil showed a diverse archaeal community with both novel *amo*A sequences and *amo*A sequences similar to *C. symbiosum* and *N. maritimus* (Turque et al. 2010). The authors further suggest, based on comparing the archaeal communities of sponges from high and low human-impacted areas, that AOA in the sponges may influence the fitness of sponges near polluted areas (Turque et al. 2010). We have shown here that diverse Archaea are present in *X. muta*, which has not been previously described in this sponge, and provides additional support for the presence of AOA in *X. muta* that has been suggested in previous studies (Lopez-Legentil et al. 2010; C. L. Fiore unpubl. data).

The significant differences in the prokaryotic community composition observed between the seawater and sponges samples is consistent with previous studies (Hentschel et al. 2002; Lee et al. 2010; Fan et al. 2012), and supports the existence of sponge-specific prokaryotic communities that have been observed in other studies (Taylor et al. 2007; Simister et al. 2012; Taylor et al. 2013). We also hypothesized that there would be location-specific differences in the prokaryotic community of *X. muta*. Based on the results of the ANOSIM and UniFrac p-test we observed significant differences in the prokaryotic community for both the sponge and the water column samples between locations. The differences we observed are important given that several studies have documented that the symbiotic prokaryotic communities of sponges from different marine habitats are similar (Taylor et al. 2007; Schmitt et al. 2011; Simister et al. 2012), and one previous study that characterized sponge prokaryotic communities from different locations in the Red Sea also did not find significant differences between locations (Lee et al. 2010). The sponges in the current study do in fact have similar taxonomic composition with several sponge-specific groups, but differ in the abundance of these taxonomic groups. Similarly, seawater samples are also more similar to each other than to sponges, but differ in the abundance of their shared taxonomic groups, and contain none of the sponge-specific groups observed in this study. There is apparently enough distance and/or differences in habitat between the three locations that selects for differences in the symbiotic community of *X. muta* from these locations. However, the biological significance of these differences is unknown.

While several studies have reported similar microbial communities from sponges in different marine habitats, one recent characterization of sponge prokaryotic communities in 32 sponge species provided support for a tropical clade of sponge symbiotic prokaryotes, indicating that subpopulations of sponge symbionts may be selected for based on specific environmental factors (Schmitt et al. 2011). In this study the bacterioplankton communities differed by location, and environmental factors have been shown to influence bacterioplankton community structure (Martiny et al. 2006; Pommier et al. 2007). Furthermore, given the proposed model for sponge symbiont transmission, where both vertical (parent to offspring) and horizontal (acquired from the environment) contribute to the community composition (Schmitt et al. 2008; Webster et al. 2010), it is possible that there is a common prokaryotic community that is vertically transmitted in *X. muta,* but that bacterioplankton from the water column also contribute to the symbiotic community. Some of the recovered prokaryotic 16S rRNA gene sequences may be from transient organisms passing through the sponge as it was feeding at the time of sampling but the differences between the sponge and seawater community are so distinct, it is likely that transient prokaryotes comprise a negligible proportion of the recovered gene sequences.

Insight into the differences in the prokaryotic community of *X. muta* and seawater between locations is provided by the results of the OTU category significance test. Synechococcaceae and Alphaproteobacteria OTUs were more abundant in the LC sponges, both of which contain common bacterioplankton members, although these OTUs were particularly more abundant in sponges than seawater. FL and LSI sponges had similar abundances of OTUs that were significantly more abundant in sponges, and the lineages of these OTUs were taxa that are known to contain sponge symbionts. To some extent the OTU abundances in the sponges followed the same trend of abundance in the seawater samples. For example, OTU 1790, classified as *Poribacteria*, is significantly more abundant in LSI and FL sponges relative to the LC sponges, and within the water column samples OTU 1790 is most abundant in the LSI samples followed by LC and FL. The extent that regional environmental factors (e.g., region-specific oceanography, temperature, nutrients) or localized factors (i.e., environment immediately surrounding individual sponges) might play a role in structuring the prokaryotic communities of *X. muta* is still unknown.

Many of the OTUs identified by the OTU significance test were observed again in the comparison of overlapping OTUs between the three locations, again providing support for differences between location being driven by abundance rather than composition. LC appeared to be the most dissimilar from the other two locations and the taxa that differ the most between locations provides some insight into what may be driving these differences. LC is more geographically separated from FL and LSI and located in the central Caribbean. Here, it is subject to a different oceanographic regime than FL and LSI and the OTUs within the Synechococcaceae and Oceanospirillales are more abundant in the LC sponges, which may be representative of this more “oceanic” site. It is also these OTUs that differ the most within the LC sponges and there may even be within site differences in nutrients or abiotic factors that can influence the symbiotic community structure. That *Poribacteria* OTUs are more abundant in FL and LSI is intriguing and perhaps the mixotrophic lifestyles of these bacteria allow them to flourish preferentially at these two locations.

While 16S rRNA gene sequences cannot be used to infer functionality, there are a few exceptions where a strong connection between taxonomy and function are well established such as with the nitrifying bacteria *Nitrospira* (Bayer et al. 2008) and potentially the Chromatiales and Syntrophobacteraceae, which are well known to be involved in sulfur cycling (Hoffmann et al. 2005; Taylor et al. 2007 and references therein). All of these taxonomic groups were recovered from *X. muta* in this study, and nitrification in particular has been documented for *X. muta* previously (Southwell et al. 2008; Fiore et al. 2013). The potential for anaerobic processes has also recently been documented in *X. muta* based on nutrient analyses (Fiore et al. 2013); however, additional research will be needed to link the taxonomic diversity of the symbiotic microbial communities in sponges with their functional diversity.

## Supporting Information

**Figure S1.** Rarefaction curves for the OTU (97% similarity) sequences of 16S rRNA genes from *X. muta* and seawater based on observed species estimates (A). Water samples are abbreviated H20 and sponge samples are abbreviated Xm. Samples are further labeled by location (FL, Florida Keys; LC, Little Cayman; LSI, Bahamas) and replicate number (1–3). Also shown are the average rank abundance curves based on the number of sequences in each OTU for *X. muta* (“Sponge”) and for seawater (“Seawater”) (B).

**Figure S2.** The ten most abundant OTUs that are unique to each location. For LC there was only one unique OTU. “Bacteria” indicates that an unidentified bacteria was the closest match. OTUs are ordered in decreasing abundance for each location and taxonomic names shown are the lowest taxonomic assignment by RDP. Locations are represented by colors shown by the legend and a second legend provides a scale for OTU abundance. The OTU number (identity) is shown in parentheses for each OTU, and OTU abundance refers to the number of sequences within that OTU. FL, Florida Keys; LC, Little Cayman; LSI, Bahamas.

**Figure S3.** The ten most abundant OTUs that are unique to individual sponges from LC. OTUs are ordered in decreasing abundance for each individual and taxonomic names shown are the lowest taxonomic assignment by RDP. “Bacteria” indicates that an unidentified bacteria was the closest match. Individual sponges are represented by colors shown by the legend and a second legend provides a scale for OTU abundance. The OTU number (identity) is shown in parentheses for each OTU, and OTU abundance refers to the number of sequences within that OTU. FL, Florida Keys; LC, Little Cayman; LSI, Bahamas.

**Table S1.** Results of the OTU category significance test in QIIME, the corrected *P*-value, means at each location, and a consensus lineage is given for each significant OTU (listed by OTU number).
