# Supplementary figures and images for: Symbiotic prokaryotic communities from different populations of the giant barrel sponge, Xestospongia muta
Source: Microbiologyopen. 2013 Sep 30;2(6):938–52. doi: 10.1002/mbo3.135 (PMC3892340; doi:10.1002/mbo3.135)

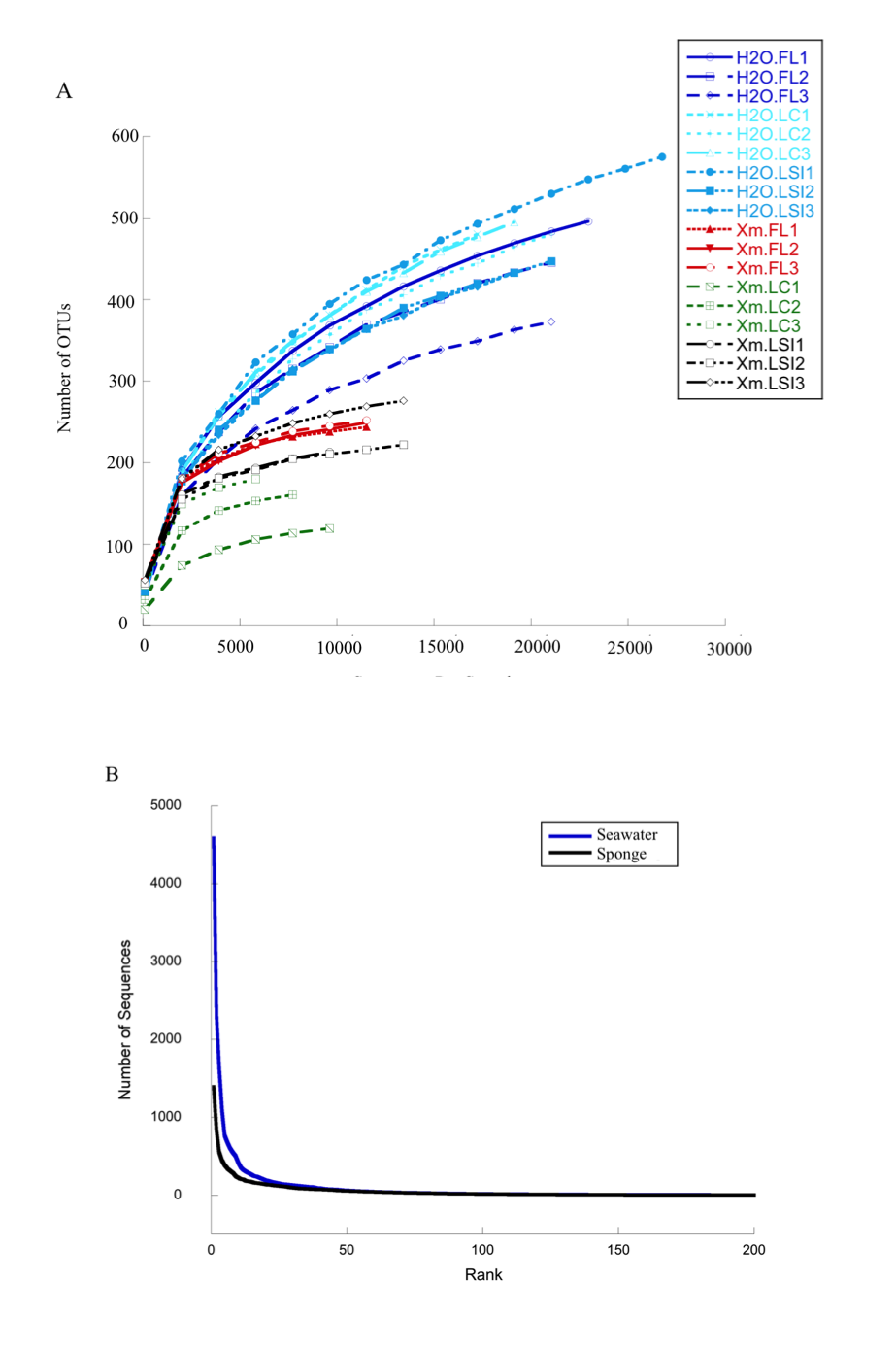

Supplement: Supplementary file 1 [file mbo30002-0938-SD1.tif]

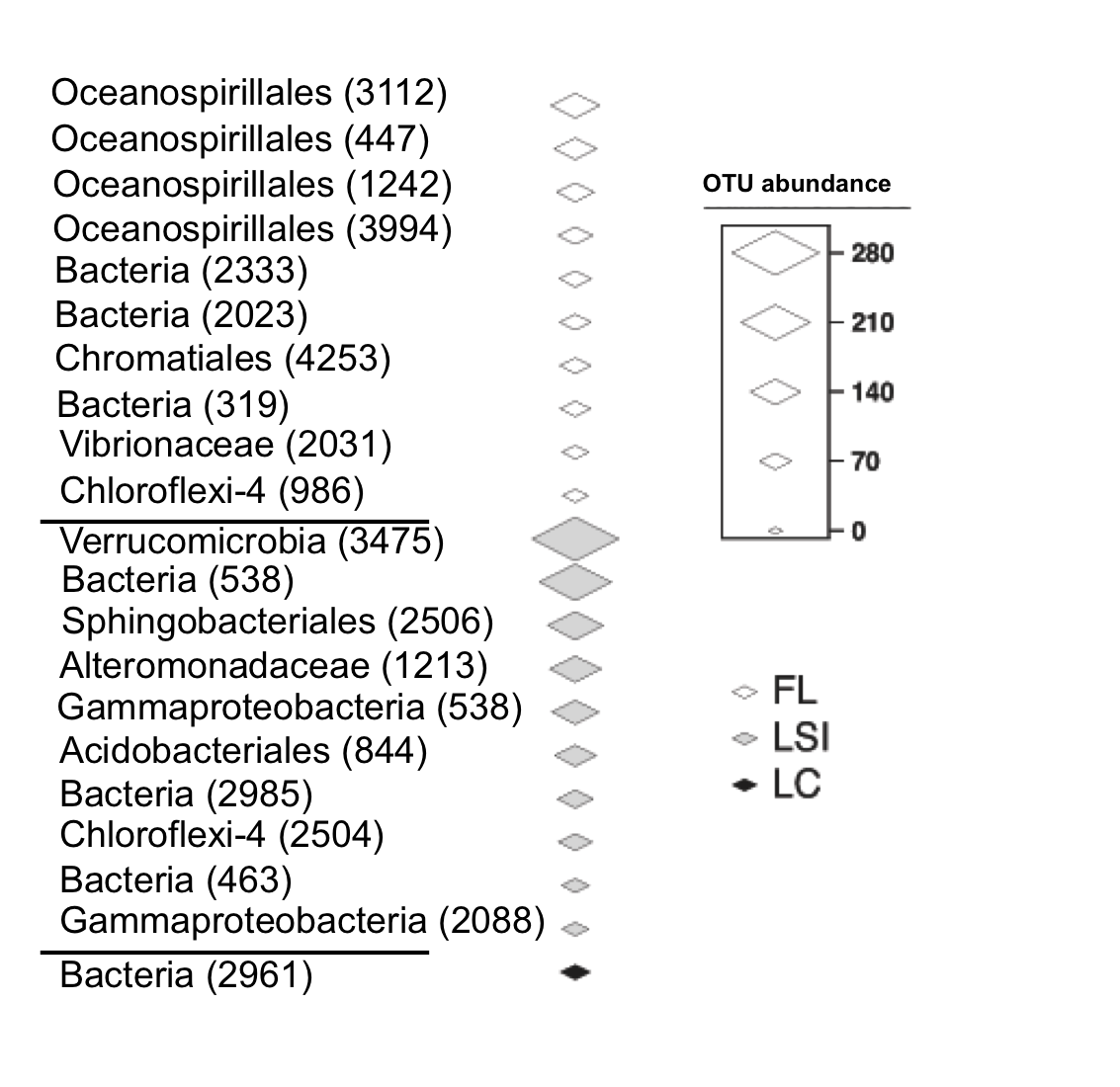

Supplement: Supplementary file 2 [file mbo30002-0938-SD2.tif]

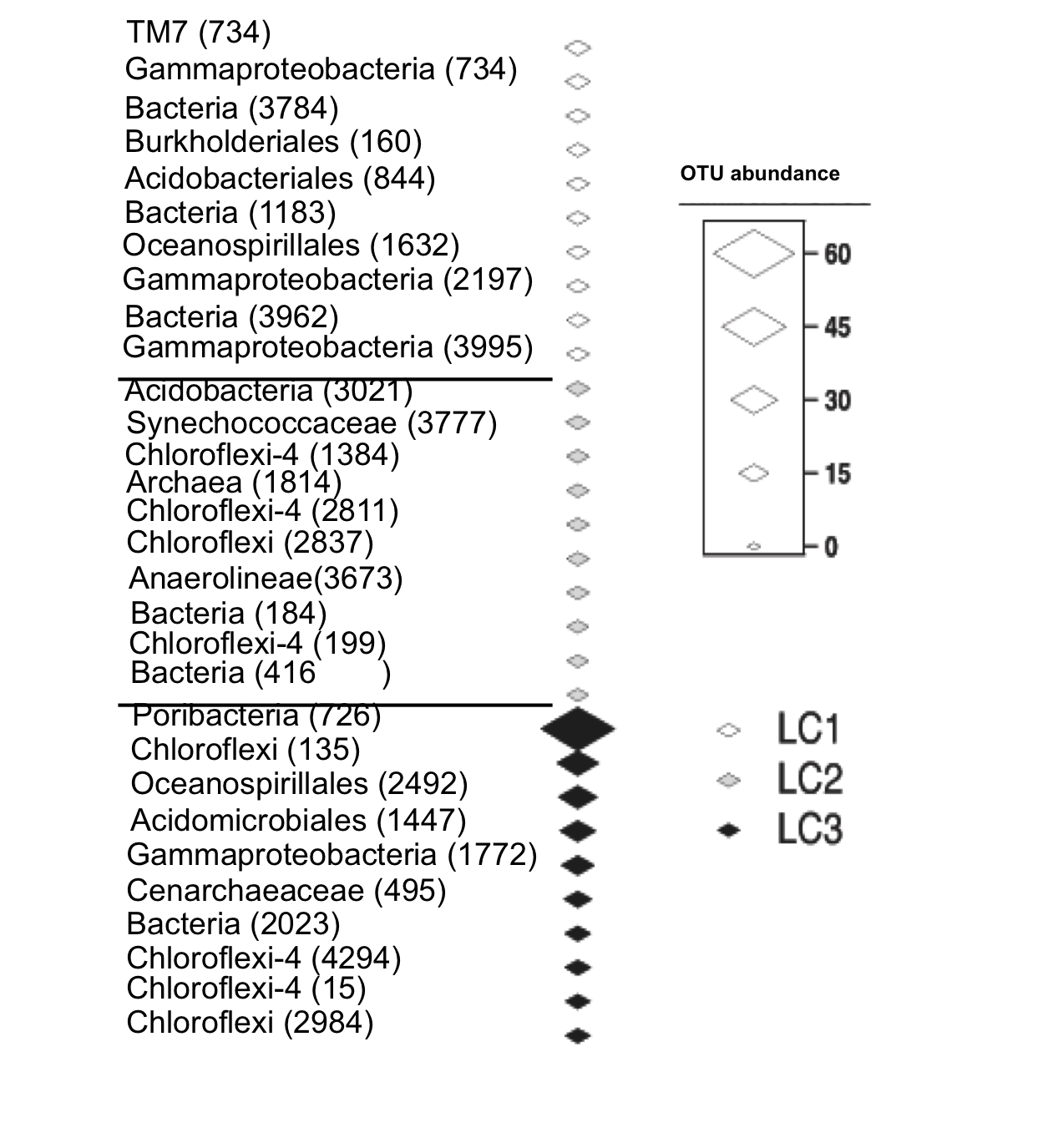

Supplement: Supplementary file 3 [file mbo30002-0938-SD3.tif]
