# Supplementary material for: Symbiotic prokaryotic communities from different populations of the giant barrel sponge, Xestospongia muta
Source: Microbiologyopen. 2013 Sep 30;2(6):938–52. doi: 10.1002/mbo3.135 (PMC3892340; doi:10.1002/mbo3.135)
Supplement: Supplementary file 4 [file mbo30002-0938-SD4.docx]

Table S1. Results of the OTU category significance test in QIIME, the corrected p-value, means at each location, and a consensus lineage is given for each significant OTU (listed by OTU number). Relative abundance (more abundant in sponges or water samples) is also given. FL = Florida Keys, LC = Little Cayman, LSI = Bahamas.
